# Native ambient mass spectrometry of intact protein assemblies directly from *Escherichia coli* colonies†

**DOI:** 10.1039/d2cc02085h

**Published:** 2022-05-24

**Authors:** Yuying Du, Robin C. May, Helen J. Cooper

**Affiliations:** School of Biosciences, University of Birmingham Edgbaston Birmingham B15 2TT UK h.j.cooper@bham.ac.uk

## Abstract

Here, we demonstrate that by combining electroporation with native ambient mass spectrometry, it is possible to detect intact non-covalent protein complexes directly from bacterial colonies growing on agar. Homodimers HdeA and HdeB were identified, together with the 50 kDa Mn-bound superoxide dismutase homodimer, in addition to some previously undetected monomeric proteins.

## Abstract 2

Electroporation combined with native liquid extraction surface analysis mass spectrometry allows analysis of intact protein assemblies directly from bacterial colonies.

## Graphical abstract

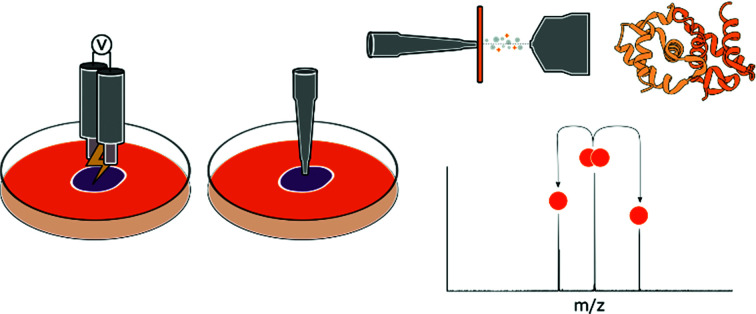

Liquid extraction surface analysis (LESA)^1^ mass spectrometry (MS) is an ambient mass spectrometry technique which combines liquid microjunction sampling with nano-electrospray mass spectrometry, and is particularly suitable for the analysis of intact proteins. LESA MS has been demonstrated for the analysis of proteins for a range of substrates, including dried blood spots,^2^ thin tissue sections^3^ and bacterial colonies growing on agar.^4–6^ Compared with matrix-assisted laser desorption ionization time-of-flight (MALDI TOF) MS, which is recognized as the gold standard for routine identification of a wide range of microorganisms,^7,8^ LESA MS focuses on the identification of intact bacterial proteins rather than spectral matching for species identification. Other ambient MS approaches that have been applied to bacteria, albeit for the analysis of small molecules, include rapid evaporative ionization mass spectrometry (REIMS),^9^ laser ablation electrospray ionization (LAESI),^10,11^ paperspray mass spectrometry^12^ and nanospray desorption electrospray ionization (nano-DESI).^13^

In terms of protein identification, analysis can either be “top-down”, in which the intact protein ion is fragmented in the mass spectrometer, or “bottom-up”, in which proteolytic peptides are subjected to MS analysis. The advantage of top-down MS is that all information relating to primary sequence and post-translational modifications are retained, unlike the bottom-up approach where information relating to the presence of single nucleotide polymorphisms or connectivity between post-translational modifications may be lost. Our work to date on LESA MS of bacteria has made use of denaturing LESA solvents based on aqueous organic mixtures.^5,14^ The use of these has been necessary to breach the cell wall. A limitation of this approach is that any structural information is lost as the proteins are unfolded. As protein structure and function are intricately linked, there is a drive to develop analytical methods capable of obtaining structural information, *e.g.*, protein assembly stoichiometry, protein-ligand binding, directly from the bacteria.

Another branch of MS, native mass spectrometry,^15^ allows the analysis of tertiary and quaternary protein structure. Proteins are electrosprayed from solutions designed to mimic native conditions, and intra- and intermolecular non-covalent interactions present in solution are maintained in the gas-phase. We have demonstrated that native mass spectrometry may be coupled with LESA MS and nano-DESI MS for the analysis of protein assemblies and complexes directly from tissue.^16,17^ Native LESA MS generally offers higher sensitivity but lower spatial resolution for mass spectrometry imaging than nano-DESI MS due to the larger sampling area. Sun and co-workers have demonstrated native mass spectrometry of *E. coli* lysates by integrating size exclusion chromatography and capillary zone electrophoresis.^18^ Huang and co-workers introduced an approach termed “in-cell” MS in which suspensions of bacteria are subjected to on-line electroporation, releasing endogenous proteins for introduction to the mass spectrometer.^19^ Electroporation is the process by which cell membranes are made permeable, either reversibly or irreversibly, through the application of an electric field,^20–22^ and has found applications in both the delivery of exogenous molecules into cells and the extraction of molecules from cells.^23^ For the latter, it has been shown that higher molecular weight molecules such as proteins present a greater challenge than those with lower molecular weight.^24^ Nevertheless, complexes of the protein calmodulin overexpressed in *E. coli* have been observed by the in-cell MS approach.^19^

We recently designed and built an electroporation device for integration with the LESA MS workflow.^14^ In that work, our aim was analysis of intact proteins in yeast colonies. Yeast had proved resistant to LESA MS, even with the use of harsh solvents, due to its rigid chitinous cell wall, but electroporation resulted in rapid release of intact proteins. A feature of electroporation is that it is associated with minimal heating,^22^ which led us to hypothesise that by combining electroporation with native-like LESA solvents, it would be possible to perform native ambient mass spectrometry, *i.e.*, obtain structural information, directly from living bacterial colonies.

Three workflows were investigated (summarised in Fig. S1, ESI†). In the first, a small volume (2 μl) of 200 mM ammonium acetate was pipetted onto the bacterial colony, which was then subjected to electroporation followed by LESA sampling. The rationale behind addition of ammonium acetate was avoidance of air breakdown during electroporation. In the second workflow, 2 μl of 200 mM ammonium acetate was deposited onto the colony before LESA sampling, *i.e.*, the electroporation step was omitted. In the third, the bacterial colony was subjected to LESA sampling only. The success rate, defined as the number of sampling events that led to mass spectra containing peaks corresponding to proteins in relation to the total number of sampling events, was 77% for addition of solvent and electroporation, 14% for addition of solvent with no electroporation and 31% for no solvent addition and no electroporation. See Table S1, ESI.†

Initial mass spectrometry experiments were performed in the mass range *m*/*z* 900–3000, with no source collision energy. Fig. 1 shows a representative native LESA mass spectrum obtained following electroporation of an *E. coli* K12 colony. Twenty-two proteins were detected, of which eleven were identified by tandem mass spectrometry (MS/MS). See Table S2, ESI† for a summary of protein assignments. Full details are provided in Protein Identification, ESI.† Eight of the proteins identified (HdeA, HdeB, CspE, Acyl carrier protein, YibT, Hpr, PsiF and YgiW) were previously undetected by LESA MS. Among those proteins, HdeA (monomer), HdeB (monomer), CspE and Hpr have previously been identified by MALDI top-down MS of cell lysates of various strains of *E. coli*.^25^ HdeA, HdeB, YibT and YgiW were identified in a bottom-up LC-MS proteomics study of outer membrane vesicles obtained from a range of *E. coli* strains.^26^ Acyl carrier protein has been identified in a bottom-up MALDI study.^27^

**Fig. 1:**
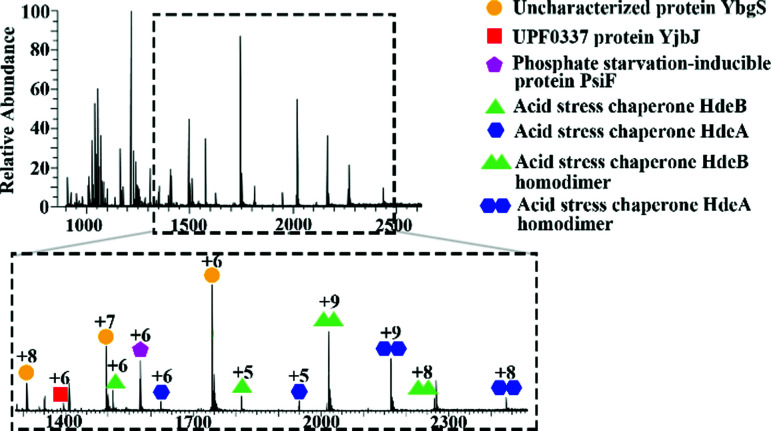
Representative mass spectrum acquired by native LESA MS following electroporation of *E. coli* K12 colony.

Subsequent experiments were performed in a higher mass range (*m*/*z* 1500–4000) with 80% source fragmentation energy, resulting in identification of two further proteins, including the monomeric antigen 43 α chain (∼49.8 kDa, see Fig. S2 and S3, ESI†) which has not previously been detected by LESA MS, although has been identified in bottom-up studies.^28–30^

In addition to monomeric proteins, three protein assemblies were identified, corresponding to the homodimers HdeA, HdeB and Mn-bound superoxide dismutase. HdeA and HdeB are acid stress chaperones which restrict aggregation of denatured periplasmic proteins in acidic environments.^31^ HdeA was detected as both the monomer (∼9.7 kDa, charge states 4+ to 6+) and homodimer (∼19.5 kDa, charge states 8+ to 9+) as indicated in Fig. 1. Fig. 2 shows the identification of the intact homodimer. Tandem mass spectrometry by higher energy collision dissociation (HCD)^32^ of the 9+ precursor ions (*m*/*z* 2165.2) resulted in detection of monomer subunits in charge states 5+ and 4+. The abundance of the monomer subunits increases with HCD collision energy (Fig. 2a). HCD of the 5+ monomer ions (32% normalised collision energy, NCE) results in detection of sequence fragments allowing the protein identity to be confirmed (Fig. 2b and c). The homodimer of HdeA, along with the homodimer of Hpr (not observed here) have previously been identified from *E. coli* lysates in native proteomics experiments which coupled size exclusion chromatography with capillary zone electrophoresis MS.^18^

**Fig. 2:**
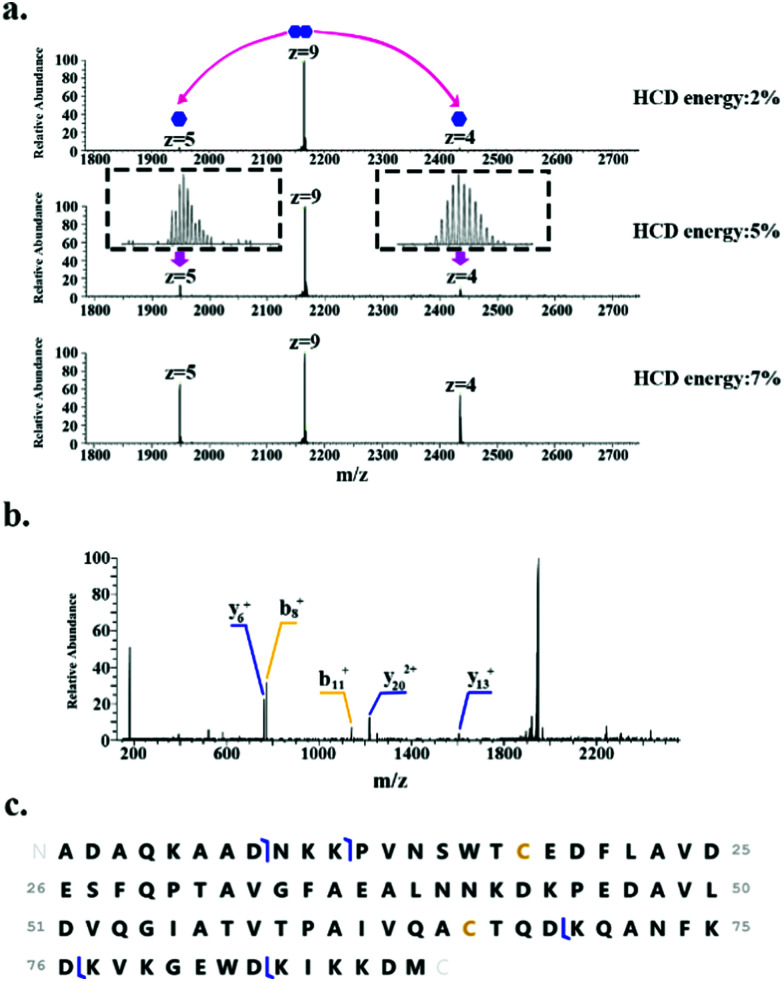
Identification of the HdeA homodimer. (a) HCD MS/MS of 9+ precursor ions (*m*/*z* 2165.2 ± 4) results in dissociation of dimer to monomer (5+ and 4+ charge states). (b) HCD MS/MS of 5+ monomer ions (*m*/*z* 1948.6). (c) Summary of sequence fragments observed.

HdeB was detected as both the monomer (∼9.1 kDa, charge states 4+ to 6+) and homodimer (∼18.1 kDa, charge states 8+ to 9+). Fig. 3 shows the identification of the homodimer. HCD of the 8+ precursor ions (*m*/*z* 2266.6) results in the observation of monomer subunits in charge states 5+, 4+ and 3+ (Fig. 3a). MS/MS of the monomer was obtained for identification (Fig. 3b) and sequence fragments are shown in Fig. 3c. The fragments observed confirm the identity of the protein and suggest the presence of a disulfide bond between Cys10 and Cys58. Confirmation of the disulphide bond was obtained by electron transfer dissociation with higher-energy collision dissociation (EThcD)^33,34^ of the 5+ monomer ions (*m*/*z* 1813.5). See Table S3, ESI.†

**Fig. 3:**
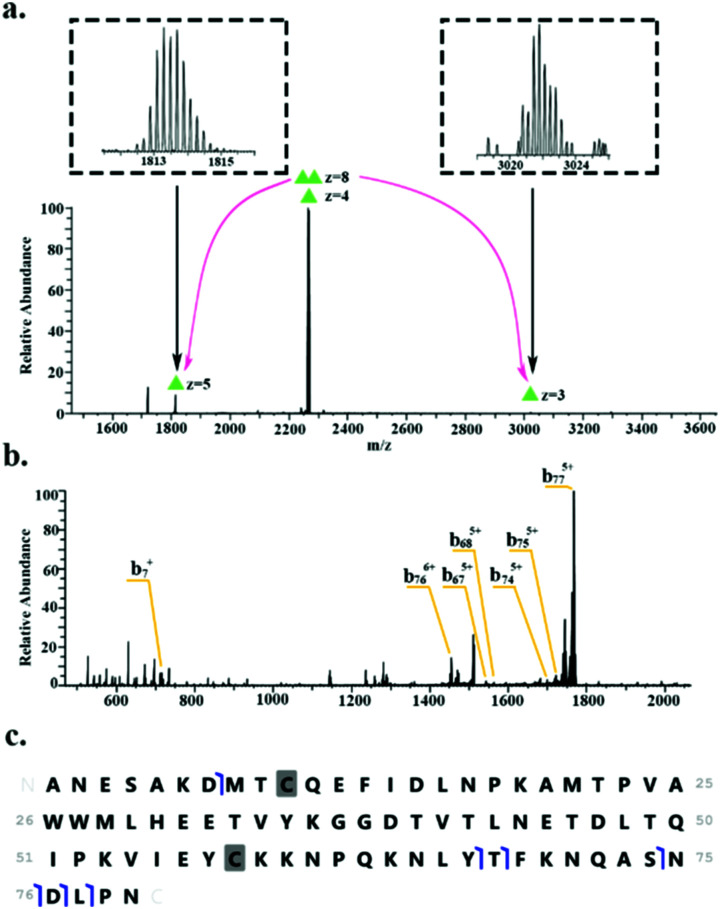
Identification of the HdeB homodimer. (a) HCD MS/MS (35% NCE) of 8+ precursor ions (*m*/*z* 2266.6 ± 4) results in dissociation of dimer to monomer (5+ and 3+ charge states). (b) HCD MS/MS of 5+ monomer ions (*m*/*z* 1813.5). (c) Summary of sequence fragments observed.

Superoxide dismutase was detected as a dimer (∼49.8 kDa) in which both subunits are bound to two Mn^2+^ ions. Fig. 4a shows a full scan mass spectrum with the peak corresponding to the 14+ charge state of the dimer indicated. The stoichiometry of the complex was confirmed by MS/MS (HCD at 28% NCE) which resulted in dissociation of the dimer to its monomeric subunits in charge states 5+ and 9+ (Fig. 4b). Asymmetric partitioning of the metal ions amongst the subunits was observed following HCD. Further increase in HCD energy resulted in detection of sequence fragments allowing the protein identity to be confirmed (see ESI†).

**Fig. 4:**
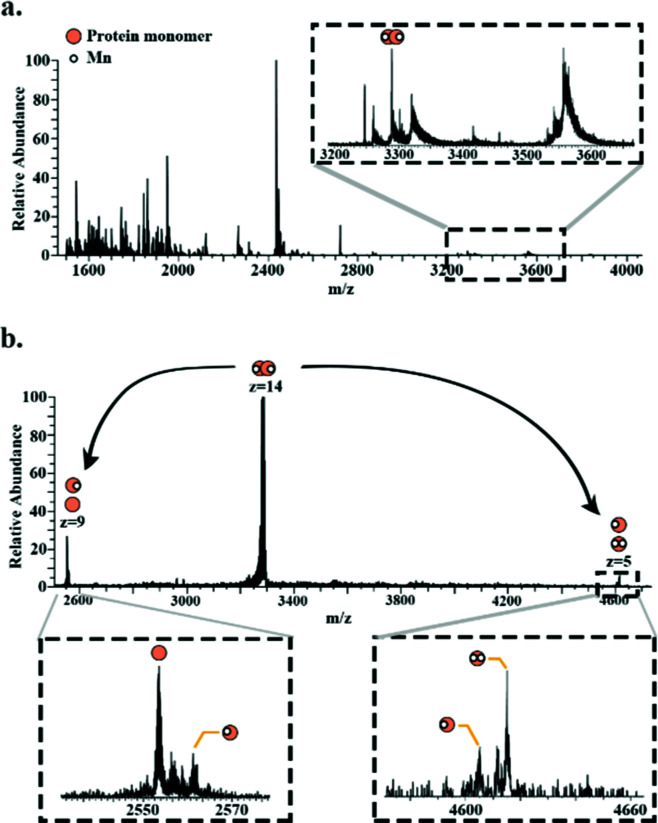
Native LESA mass spectrum of Mn-bound superoxide dismutase dimer. (a) Full scan mass spectrum with enlarged *m*/*z* region showing the peak corresponding to superoxide dismutase dimer. (b) HCD of the 14+ precursor ions (*m*/*z* 3289.5 ± 6) results in dissociation of the dimer to monomer (9+ and 5+ charge state) with differing numbers of Mn^2+^ bound.

In summary, we have demonstrated that by combining electroporation with native LESA MS, it is possible to detect and identify intact protein assemblies up to 50 kDa directly from colonies of *E. coli* growing on a solid substrate. The stoichiometry of the protein assemblies and the identities of the proteins were confirmed by HCD MS. Although it was possible in some cases to detect proteins in the absence of electroporation, this could not be achieved reliably (success rate 14–31%).

YD is funded by a Darwin Studentship. HJC is funded by EPSRC (EP/S002979/1). The authors thank Dr Emma Sisley for helpful discussions. The Orbitrap Eclipse mass spectrometer used in this work was funded by BBSRC (BB/S019456/1). Supplementary data supporting this research is openly available from: https://doi.org/10.25500/edata.bham.00000841.

## Conflicts of interest

There are no conflicts of interest to declare.

## Supplementary Material

CC-058-D2CC02085H-s001
