# Supplementary material for: Native ambient mass spectrometry of intact protein assemblies directly from Escherichia coli colonies
Source: Chem Commun (Camb). 2022 May 24;58(48):6857–60. doi: 10.1039/d2cc02085h (PMC9196203; doi:10.1039/d2cc02085h)
Supplement: CC-058-D2CC02085H-s001 [file CC-058-D2CC02085H-s001.pdf]

Electronic Supplementary Material (ESI) for ChemComm.
This journal is © The Royal Society of Chemistry 2022

Supporting Information

Native ambient mass spectrometry of protein complexes directly from *Escherichia coli* colonies

Yuying Du, Robin C. May and Helen J. Cooper

School of Biosciences, University of Birmingham, Edgbaston, Birmingham B15 2TT, UK.

Table of contents:

Experimental details

Figure S1. Comparison of workflows for native ambient mass spectrometry of bacterial colonies.

Table S1. Comparison of the success rates of the three workflows

Table S2. Proteins identified following electroporation and native LESA top-down mass spectrometry of E. coli K-12 colonies

Table S3. EThcD MS2 sequence cleavage information of Hde B

Figure S2. Identification of protein antigen 43 alpha chain

Figure S3. Sequence coverage diagram of antigen 43 alpha chain

Protein identification

Experimental details:

Materials. LB broth (yeast extract (VWR, Lutterworth, UK), peptone (Sigma-Aldrich, Gillingham, UK) and sodium chloride (Fisher Scientific, Loughborough, UK)), LB agar (LB broth with added bacteriological agar) were prepared for culturing bacterial species. Bacterial strains E. coli K12 were obtained from Willem van Schaik (Institute of Microbiology and Infection (IMI), University of Birmingham, UK). Ammonium acetate as purchased from SigmaAldrich (Gillingham, UK). MS-grade water was purchased from Fisher Scientific (Loughborough, U.K.).

Bacterial Colonies. 1 µL of *E. coli* K12 frozen glycerol stock was resuspended in 5−10 mL of liquid broth. After being incubated at 37°C 200 rpm shaking condition in the dark for approximate 18 hours, liquid culture was inoculated onto solid LBA medium in 6 cm diameter Petri dishes. Bacteria were incubated at 37 °C overnight.

Electroporation and Surface sampling. Electroporation was performed on the colonies using the home-built device described by Kocurek et al[1]. Prior to electroporation, 2µL of 200mM ammonium acetate was pipetted onto the surface of the colony. The lifting lever was adjusted till the electrodes just touched the colony. Electroporation parameters were: voltage applied = 3 kV, field strength = 15 kV/cm, number of pulses = 30, pulse length = 20us.

LESA sampling was performed by use of a TriVersa NanoMate robotic pipet system (Advion, Ithaca, NY) controlled by the advanced user interface (AUI) feature of the TriVersa NanoMate ChipSoft Manager software 8.3.3. In brief, 3 µL of 200mM ammonium acetate were aspirated from the solvent well of which 2 µL were dispensed onto the colony at a location between the imprints of the electroporator electrodes. The height was set such that the pipette tip just touched the colony. The liquid microjunction was maintained for 15 s after which 2.5 µL were reaspirated. The sample was then introduced to the mass spectrometer by nanoESI at 1.65 kV and 0.3 psi.

Mass Spectrometry. Mass spectrometry experiments were performed by use of an Orbitrap Eclipse Tribrid mass spectrometer (Thermo Fisher, San Jose, CA), equipped with the HMR^n option. The instrument was operated in positive-ion mode and "Intact Protein" mode. Initial full scan mass spectra were acquired in the 900-3000 m/z range at a resolution of 120,000 at m/z 200, with normalised automatic gain control (AGC) target set to 100%. In later experiments, full scan mass spectra were collected in the 1500-4000 m/z range at a resolution of 240,000 at m/z 200, and the source fragmentation energy was set to 80%. Precursor ions were selected for fragmentation in the linear ion trap with an isolation window of 6 m/z. HCD was performed using 5-40% normalised collision energy. Fragments were detected in the orbitrap at a resolution of 120000 at m/z 200.

PTCR experiments were performed at a resolution of 7500 at m/z 200 in positive-ion mode with 80% source fragmentation energy. Precursor ions were selected by ion trap with an isolation window of 6 m/z. The fragments were detected in the orbitrap analyzer. The reaction time was 3ms and mass range was 3000-6000 m/z.

EThcD of HdeB was performed in the 500-4000 m/z range at a resolution of 120000 at m/z 200. Precursor ions were selected by ion trap with an isolation window of 3 m/z. The fragments were detected by orbitrap analyzer. The ETD energy was 27% and HCD energy was 38%. Other parameters were the same with full scan mass spectra.

Protein Identification. Top-down protein identification was achieved by ProSightPC software, version 4.1 alpha (Thermo Fisher Scientific, Bremen, Germany). The whole organism proteome database for *E. coli* K12 were downloaded in XML format from the UniProt Web site (uniprot.org)

Uniprot ID UP000000625, 4448 protein entries). Absolute mass mode was used for identification. MS2 spectra were deconvoluted by the THRASH algorithm at a signal-to-noise ratio of 3. Considering the initial methionine cleavage and n-terminal acetylation, database was constructed as a standard top-down database. Single nucleotide polymorphisms and all available posttranslational modifications were considered with a maximum of 13 features per sequence and maximum mass of 70 kDa which has been reported previously[2]. Fragment ion tolerance was set up as 20 ppm. Protein identities were confirmed by manual analysis using Protein Prospector (http://prospector.ucsf.edu/prospector/mshome. htm).

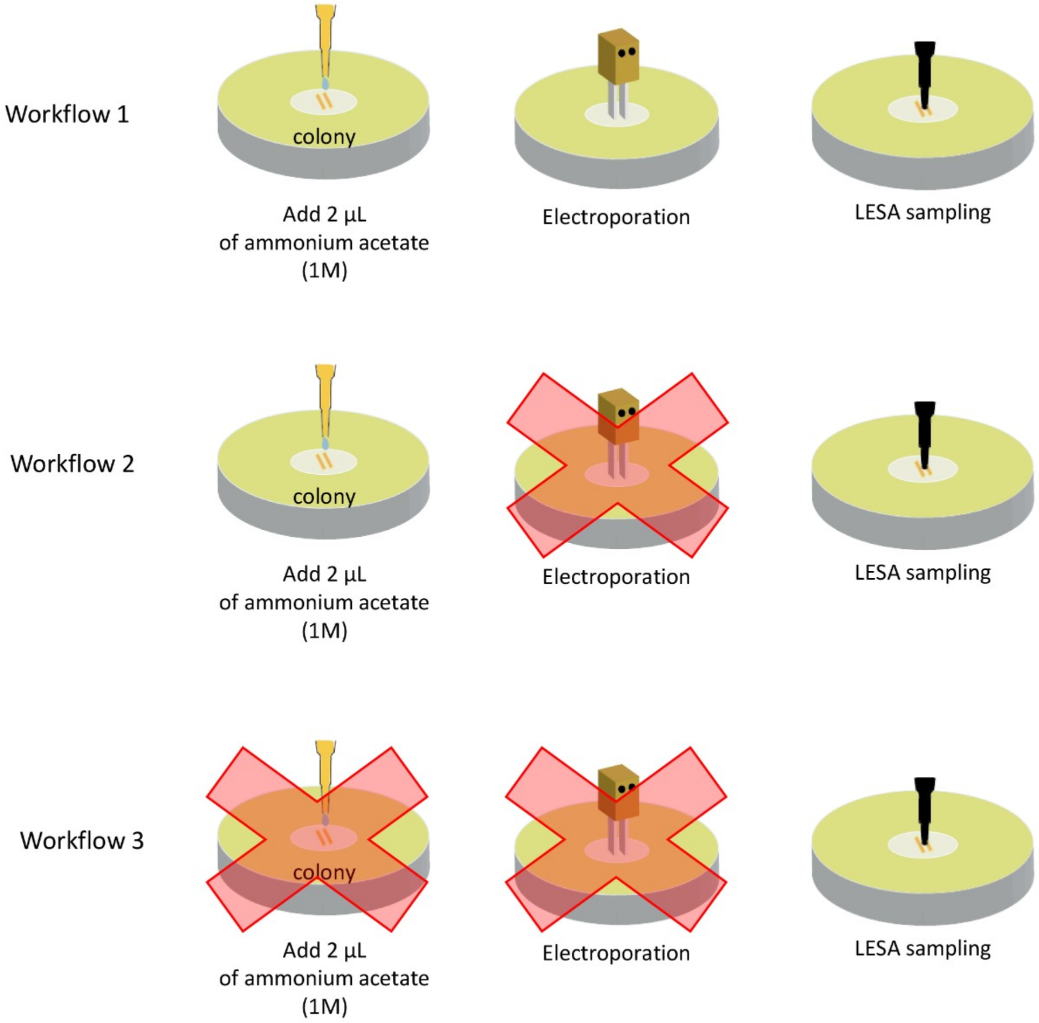

Figure S1. Comparison of workflows for native ambient mass spectrometry of bacterial colonies. Workflow 1: a) 2 µl of 200 mM ammonium acetate are deposited on the surface of the colony, b) the colony is subjected to electroporation and c) LESA sampling of the colony by use of 200 mM ammonium acetate.

Workflow 2: 2 µl of solvent are deposited on the surface of the colony followed by LESA sampling of the colony with 200 mM ammonium acetate.

Workflow 3: LESA sampling of the colony with 200 mM ammonium acetate.

Table S1. Comparison of the success rates of the three workflows

Workflow	Number of sampling events	Number of sampling events resulting in mass spectra containing protein peaks	Success rate
Workflow 1 (w/ electroporation)	35	27	77.14%
Workflow 2 (w/o electroporation)	22	3	13.64%
Workflow 3 (LESA control)	35	11	31.42%

Table S2 Summary of proteins identified following electroporation and native LESA top-down mass spectrometry of E. coli K-12 colonies

protein identity	m/z_{means}	m/z_{calc}	Δppm	charge state	MW_{calc}	remarks
Cold shock protein CspA	1454.3318	1454.7190	1.9360	+5	7267.5877	
Cold shock-like protein CspE	1466.5429	1466.5547	0.0590	+5	7327.7735	
Uncharacterized protein YibT	1310.8637	1310.8690	0.0319	+6	7859.2141	
UPF0337 protein YjbJ	1387.6714	1387.6821	0.0640	+6	8320.0923	
Acyl carrier protein	1701.6255	1701.6361	0.0526	+5	8503.1803	no methionine, no acetylation, no O3-phosphopantetheine-L-serine
	1770.4559	1769.6224	0.0958	+5	8843.1122	no methionine, no acetylation, O3-phosphopantetheine-L-serine
	1778.0382	1778.0244	0.0688	+5	8885.1222	no methionine, acetylation, O3-phosphopantetheine-L-serine
Acid stress chaperone HdeB	1812.4694	1812.8781	2.0437	+5	9059.3905	monomer
	2014.7389	2014.1979	-4.8690	+9	18118.7811	dimer
Phosphocarrier protein HPr	1519.9440	1519.9562	0.0731	+6	9113.7373	
Phosphate starvation-inducible protein PsiF	1574.9150	1587.4404	75.1527	+6	9518.6425	
Uncharacterized protein YbgS	1745.0939	1744.1006	-5.9596	+6	10458.6036	
Protein YgiW	1710.8410	1710.8553	0.1005	+7	11968.9873	
Acid stress chaperone HdeA	1947.4906	1947.9657	2.0430	+5	9734.8287	monomer
	2163.8416	2164.2953	4.0833	+9	19469.6575	dimer
Antigen 43 α chain	3557.1320	3557.1426	0.1487	+14	49758.7680	
Superoxide dismutase	3288.5097	3287.4381	-15.0024	+14	46023.1376	dimer with 2 Mn

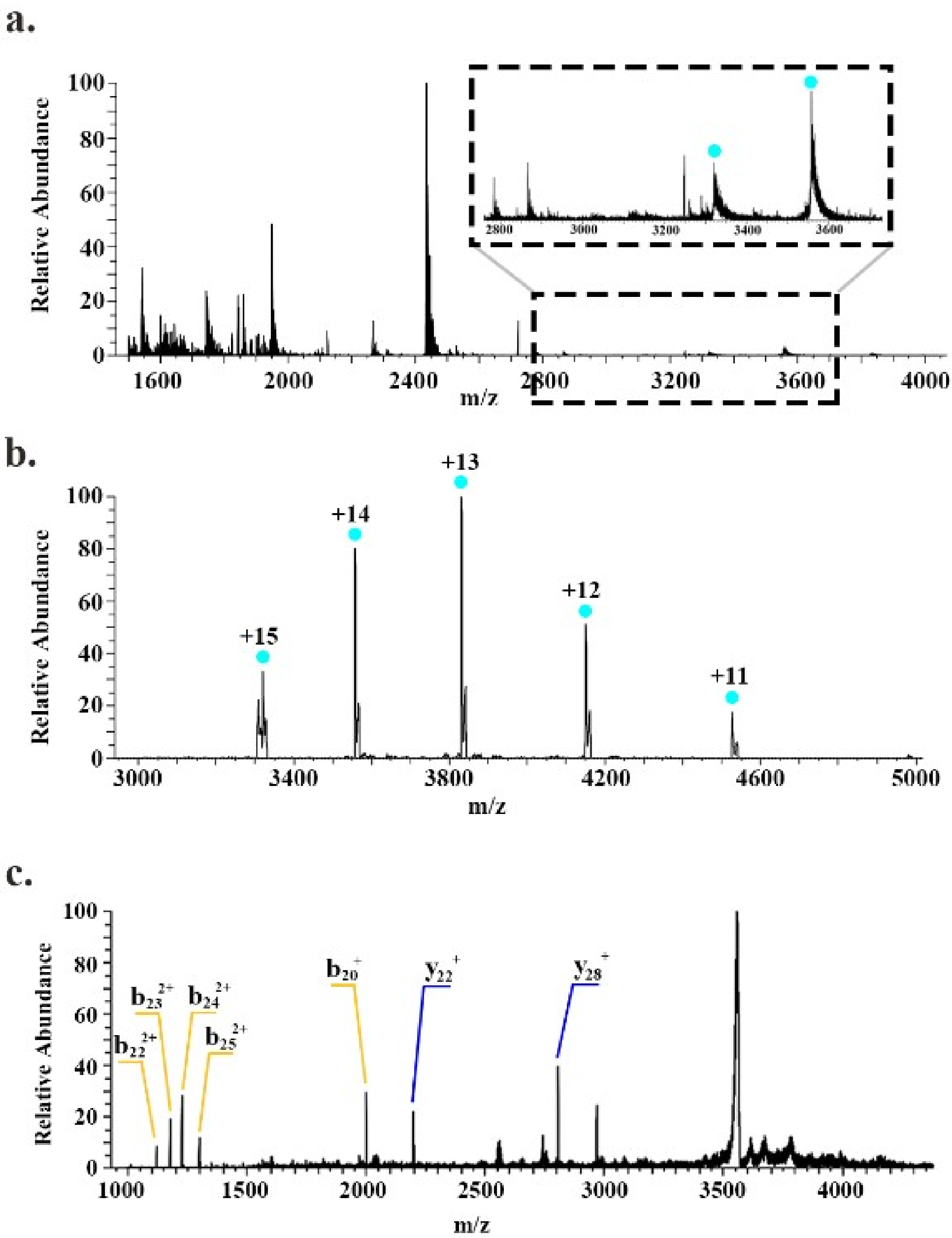

Figure S2 Identification of protein antigen 43 alpha chain **a.** Full scan mass spectrum with enlarged m/z region showing peaks corresponding to 15+ and 14+ charge states of antigen 43 alpha chain **b.** PTCR mass spectrum obtained for precursor ions with m/z 3320 (15+ charge state) with isolation window of 10 m/z. Deconvolution of this spectrum confirms the mass of the proteins to be 49758.6 Da **c.** HCD MS2 spectrum of ions centered at m/z 3556.0 (14+ charge state), identified as antigen 43 alpha chain

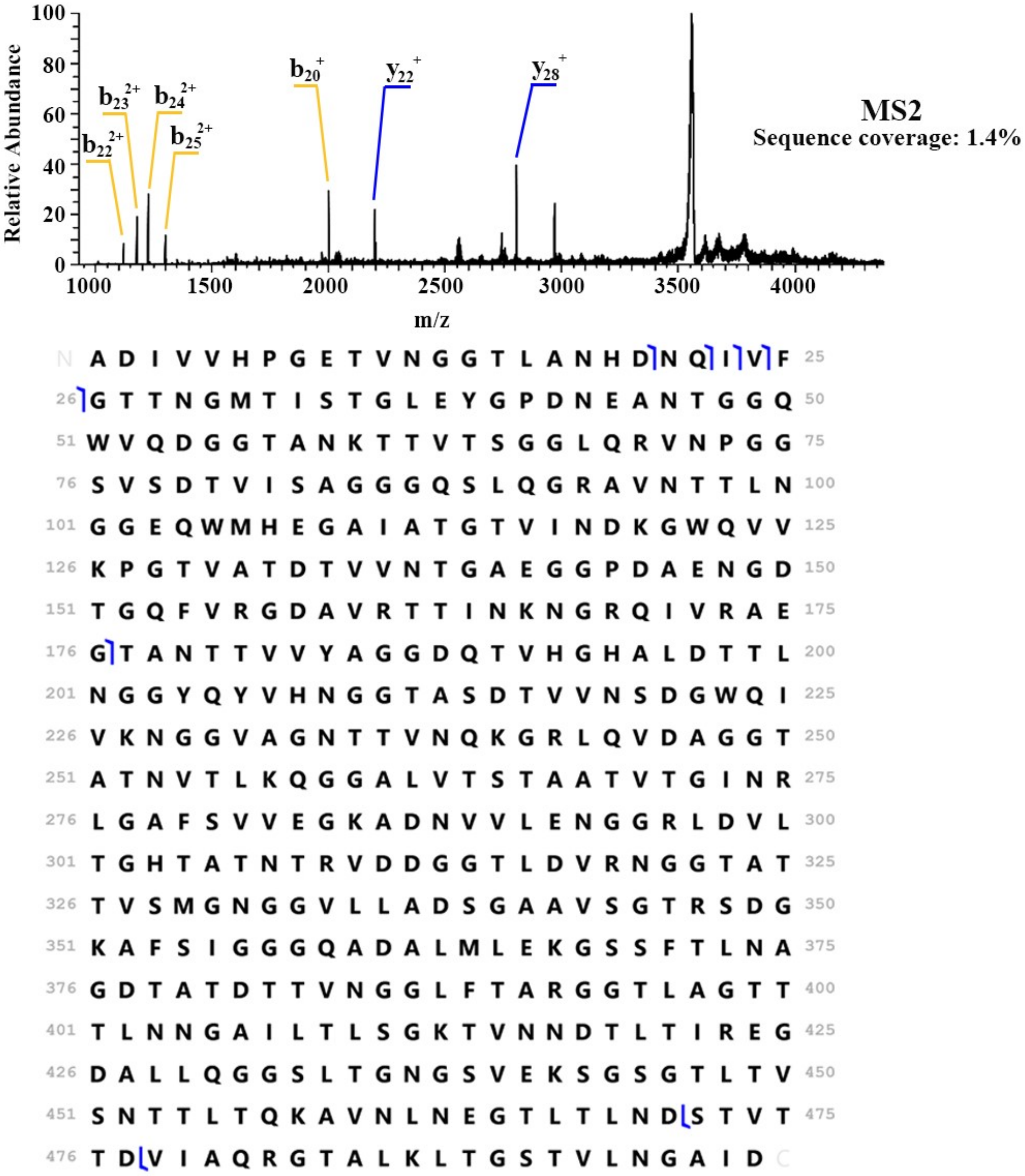

Figure S3. Sequence coverage diagram of antigen 43 alpha chain

Table S3. Fragments identified following EThcD MS/MS of Hde B. The observation of a 2 Da mass difference on the y24 fragment ions indicate the presence of the disulfide bond.

Name	Mass Monoisotopic	Theoretical Mass	Mass dif. (Da)
b27	3077.3334	3077.4230	-0.0896
c27	3094.4582	3094.4496	0.0086
c30	3475.6430	3475.6330	0.0100
z30	3518.6194	3518.8252	-0.2058
y24	2854.3316	2856.4010	-2.0694

From the information of EThcD MS2, we found that most ions matched their theoretical mass with minor mass difference.

Protein Identification

All masses listed are monoisotopic.

E. coli K12

Protein name: Cold shock protein CspA (Uniprot accession no. P0A9X9)

Charge state: +5

Observed monoisotopic mass: 7266.6590 Da

Sequence:
SGKMTGIVKWFNADKGFGFITPDDGSKDVFVHFSAIQNDGYKSLDEGQKVSFTIESGAKGPAAGNVTSL

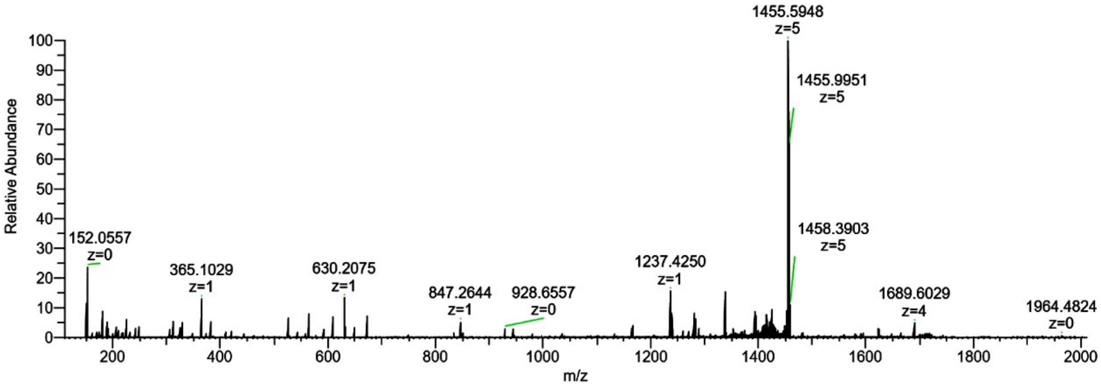

ID	Name	m/z Monoisotopic	Mass Monoisotopic	Intensity	Theoretical Mass	Error (Da)	Error (ppm)
10	b69	1454.5079	7267.5032	8.6975e+002	7267.5334	-0.0302	-4.1612
5	y66	1373.6779	6863.3531	2.4105e+002	6995.4391	-0.0711	-10.1681
10	y69	1454.5079	7267.5032	8.6975e+002	7267.5876	-0.0844	-11.6100

Protein name: Cold shock-like protein CspE (Uniprot accession no. P0A972)

Charge state: +5

Observed monoisotopic mass: 7327.7145 Da

Sequence:
SKIKGNVKWFNESKGFGFITPEDGSKDVFVHFSAIQTNGFKTLAEGQRVEFEITNGAKGPSAANVIAL

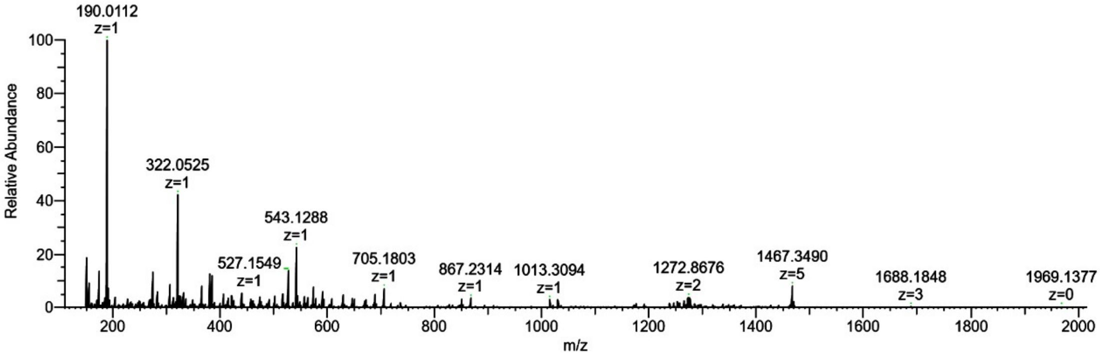

ID	Name	m/z Monoisotopic	Mass Monoisotopic	Intensity	Theoretical Mass	Error (Da)	Error (ppm)
35	b21	1135.1000	2268.1855	9.3829e+001	2399.2460	0.0388	16.1708
55	b68	1466.5446	7327.6864	4.7086e+003	7327.7192	-0.0327	-4.4693
55	y68	1466.5446	7327.6864	4.7086e+003	7327.7733	-0.0869	-11.8569

Protein name: Uncharacterized protein YibT (Uniprot accession no. Q2M7R5)

Charge state: +6

Observed monoisotopic mass: 7859.1822 Da

Sequence:
GKLGENVPLLIDKAVDFMASSQAFREYLKKLPPRNAIPSGIPDESVPLYLQRLEYYRRLYRPKQVEGQ

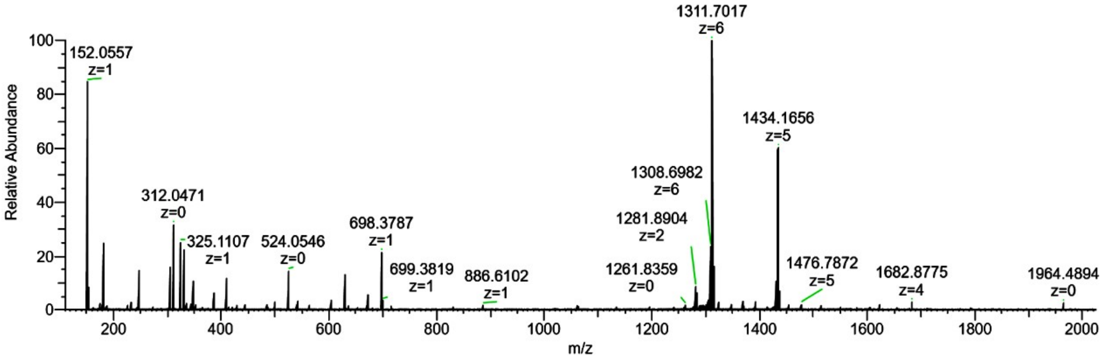

ID	Name	m/z Monoisotopic	Mass Monoisotopic	Intensity	Theoretical Mass	Error (Da)	Error (ppm)
9	b7	698.3796	697.3724	2.3303e+002	697.3759	-0.0035	-5.0446
22	y61	1433.3650	7161.7884	2.6473e+003	7161.8381	-0.0497	-6.9457
22	y61	1433.3650	7161.7884	2.6473e+003	7161.8381	-0.0179	-2.5035
11	y66	1280.0070	7673.9982	1.2113e+002	7674.0976	-0.0994	-12.9503
11	y66	1280.0070	7673.9982	1.2113e+002	7674.0976	-0.0676	-8.8046

Protein name: UPF0337 protein YjbJ (Uniprot accession no. P68206)

Charge state: +6

Observed monoisotopic mass: 8320.0283 Da

Sequence:
MNKDEAGGNWKQFKGKVKEQWGKLTDDDMTIIEGKRDQLVGKIQERYGYQKDQAEKEVVDWETRN EYRW

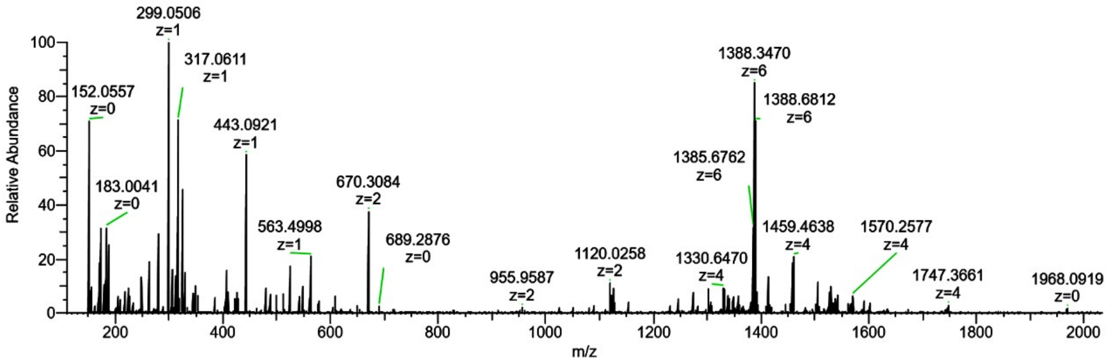

ID	Name	m/z Monoisotopic	Mass Monoisotopic	Intensity	Theoretical Mass	Error (Da)	Error (ppm)
18	b20	1152.5709	2303.1272	1.0326e+002	2303.1480	-0.0208	-9.0359
19	b21	1245.6104	2489.2062	1.3071e+002	2489.2274	-0.0212	-8.5155
23	b23	1338.1713	2674.3281	1.6043e+002	2674.3438	-0.0157	-5.8627
14	y3	524.2609	523.2536	2.0913e+002	523.2543	-0.0007	-1.3034
16	y9	670.3095	1338.6045	8.0786e+002	1338.6105	-0.0060	-4.5174
17	y17	1119.5201	2237.0256	3.1442e+002	2237.0501	-0.0245	-10.9711
26	y32	1348.6631	4042.9675	2.0521e+002	4042.9925	-0.0250	-6.1860
26	y32	1348.6631	4042.9675	2.0521e+002	4042.9925	0.0389	9.6227
20	y41	1272.6240	5086.4669	2.9557e+002	5086.5359	-0.0689	-13.5522
20	y41	1272.6240	5086.4669	2.9557e+002	5086.5359	-0.0050	-0.9868
21	y42	1301.3805	5201.4930	3.4646e+002	5201.5628	-0.0698	-13.4273
21	y42	1301.3805	5201.4930	3.4646e+002	5201.5628	-0.0059	-1.1397

22	y43	1330.1378	5316.5219	3.7821e+002	5316.5898	-0.0679	-12.7642
22	y43	1330.1378	5316.5219	3.7821e+002	5316.5898	-0.0039	-0.7425
35	y46	1412.4386	5645.7254	6.2970e+002	5645.7484	-0.0230	-4.0778
35	y46	1412.4386	5645.7254	6.2970e+002	5645.7484	0.0409	7.2431
36	y48	1458.7054	5830.7926	9.4101e+002	5830.8649	-0.0722	-12.3862
36	y48	1458.7054	5830.7926	9.4101e+002	5830.8649	-0.0083	-1.4247
38	y63	1527.3470	7631.6984	4.2986e+002	7631.8072	-0.1088	-14.2498
38	y63	1527.3470	7631.6984	4.2986e+002	7631.8072	-0.0448	-5.8751
39	y64	1541.5559	7702.7429	3.1376e+002	7702.8443	-0.1014	-13.1633
39	y64	1541.5559	7702.7429	3.1376e+002	7702.8443	-0.0375	-4.8658
40	y66	1590.3722	7946.8249	2.4096e+002	7946.9138	-0.0890	-11.1953
40	y66	1590.3722	7946.8249	2.4096e+002	7946.9138	-0.0251	-3.1526

Protein name: Acyl carrier protein (Uniprot accession no. P0A6A8)

Charge state: +5

Observed monoisotopic mass: 8503.1277 Da

Sequence:
STIEERVKKIIGEQLGVKQEEVTNNASFVEDLGADSLDTVELVMALEEEFDTEIPDEEAEKITTVQAAIDYIN GHQA

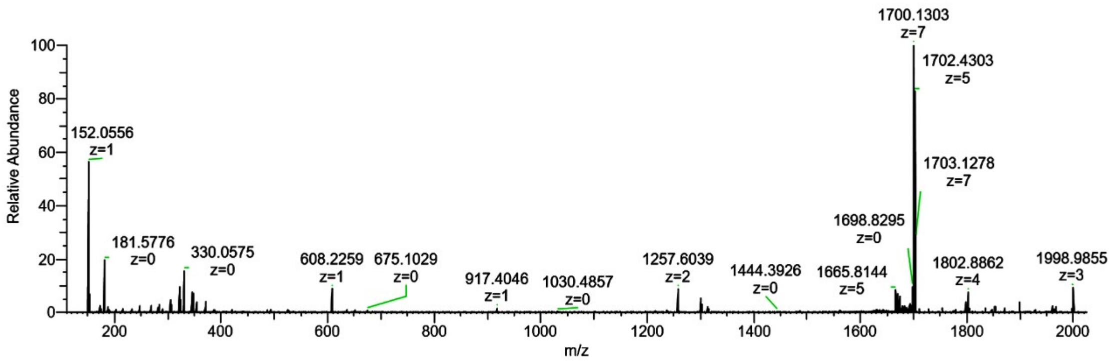

ID	Name	m/z Monoisotopic	Mass Monoisotopic	Intensity	Theoretical Mass	Error (Da)	Error (ppm)
31	b55	1997.9662	5990.8769	5.0673e+002	6164.0231	-0.0131	-2.1201
14	y12	1300.6133	1299.6060	1.3257e+002	1299.6207	-0.0148	-11.3656
13	y23	1257.0969	2512.1792	3.4712e+002	2512.2081	-0.0289	-11.5098
15	y24	1313.6418	2625.2690	7.1360e+001	2625.2922	-0.0232	-8.8226
26	y77	1701.6246	8503.0865	5.2343e+003	8503.1802	-0.0937	-11.0185

Protein name: Acyl carrier protein (Uniprot accession no. P0A6A8) (no methionine, no acetylation, O3-phosphopantetheine-L-serine)

Charge state: +5

Observed monoisotopic mass: 8843.2080 Da

Sequence:
STIEERVKKIIGEQLGVKQEEVTNNASFVEDLGADSLDTVELVMALEEEFDTEIPDEEAEKITTVQAAIDYINGHQA

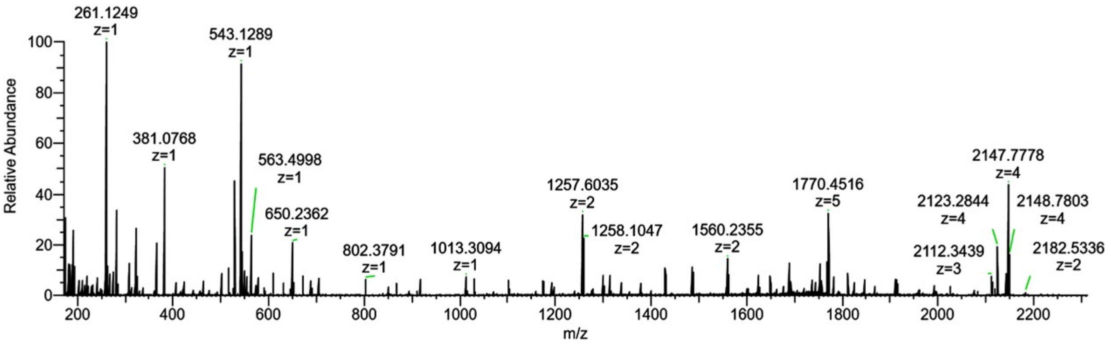

ID	Name	m/z Monoisotopic	Mass Monoisotopic	Intensity	Theoretical Mass	Error (Da)	Error (ppm)
48	b32	1736.3989	3470.7833	4.1859e+002	3601.8297	0.0521	14.4678
62	b36	1914.4641	3826.9137	3.7173e+002	3957.9993	0.0129	3.2541
34	b36	1276.6561	3826.9466	1.7368e+002	3957.9993	0.0458	11.5601
45	b44	1675.4769	5023.4088	2.7960e+002	5154.5393	-0.0320	-6.2124
49	b46	1742.8315	5225.4726	3.8254e+002	5356.6169	-0.0458	-8.5534
57	b47	1780.5515	5338.6326	6.4276e+002	5469.7009	0.0302	5.5187
24	y7	802.3782	801.3709	2.0104e+002	801.3769	-0.0060	-7.5121
26	y8	917.4032	916.3959	2.1364e+002	916.4039	-0.0080	-8.7189
29	y10	1101.5268	1100.5195	2.2875e+002	1100.5251	-0.0055	-5.0312
30	y11	1172.5614	1171.5541	2.1727e+002	1171.5622	-0.0081	-6.8857
35	y12	1300.6169	1299.6096	3.3006e+002	1299.6207	-0.0111	-8.5687
33	y23	1257.1004	2512.1862	2.1631e+003	2512.2081	-0.0219	-8.7119
36	y24	1313.6455	2625.2764	5.9121e+002	2625.2922	-0.0158	-6.0245
38	y25	1378.1635	2754.3125	2.8590e+002	2754.3348	-0.0223	-8.0785

39	y26	1428.6881	2855.3616	6.9033e+002	2855.3824	-0.0208	-7.2855
40	y27	1486.2029	2970.3912	7.4629e+002	2970.4094	-0.0182	-6.1167
41	y28	1559.7245	3117.4344	1.0461e+003	3117.4778	-0.0434	-13.9128
42	y29	1624.2465	3246.4784	6.1767e+002	3246.5204	-0.0419	-12.9175
46	y30	1688.7662	3375.5178	9.1579e+002	3375.5630	-0.0452	-13.3972
51	y31	1753.2868	3504.5590	8.7104e+002	3504.6056	-0.0465	-13.2808
58	y32	1809.8418	3617.6690	6.5612e+002	3617.6896	-0.0206	-5.6904
60	y33	1845.3636	3688.7127	4.3716e+002	3688.7267	-0.0140	-3.7929
61	y34	1910.8688	3819.7231	5.5098e+002	3819.7672	-0.0441	-11.5455
60	y34	1845.3636	3688.7127	4.3716e+002	3819.7672	0.0440	11.5210
56	y77	1769.6518	8843.2228	1.0048e+003	8843.2660	-0.0432	-4.8856

Protein name: Acyl carrier protein (Uniprot accession no. P0A6A8) (no methionine, acetylation, O3-phosphopantetheine-L-serine)

Charge state: +5

Observed monoisotopic mass: 8885.1910 Da

Sequence:
STIEERVKKIIGEQLGVKQEEVTNNASFVEDLGADSLDTVELVMALEEEFDTEIPDEEAEKITTVQAAIDYINGHQA

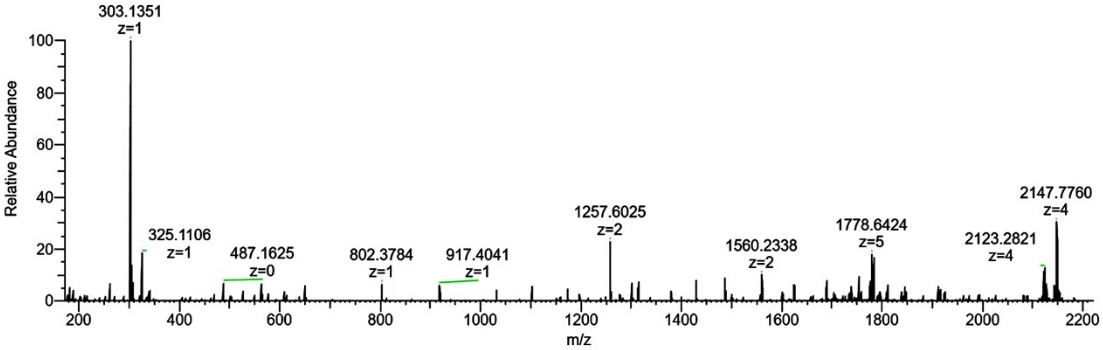

ID	Name	m/z Monoisotopic	Mass Monoisotopic	Intensity	Theoretical Mass	Error (Da)	Error (ppm)
37	b43	1656.4628	4966.3666	2.7976e+002	5097.4814	0.0112	2.1944
40	b44	1689.4968	5065.4686	5.5448e+002	5196.5498	0.0448	8.6303
46	b46	1756.8459	5267.5158	6.6559e+002	5398.6274	0.0144	2.6673
54	b47	1794.5440	5380.6103	7.1284e+002	5511.7115	0.0248	4.5040
58	b49	1880.5644	5638.6713	4.2504e+002	5769.7967	0.0006	0.1119
63	b51	1972.5999	5914.7778	4.0549e+002	6045.9077	-0.0039	-0.6392
67	b54	2087.6437	6259.9093	3.8359e+002	6391.0249	0.0105	1.6460
70	b55	2125.3496	6373.0270	1.4277e+003	6504.1089	0.0441	6.7858
13	y7	802.3814	801.3741	4.0750e+002	801.3769	-0.0028	-3.5002
14	y8	917.4073	916.4001	4.3266e+002	916.4039	-0.0038	-4.1761
15	y9	1030.4916	1029.4843	3.0225e+002	1029.4879	-0.0036	-3.5173
16	y10	1101.5278	1100.5205	4.3408e+002	1100.5251	-0.0046	-4.1635
19	y11	1172.5679	1171.5607	3.5187e+002	1171.5622	-0.0015	-1.2744
25	y12	1300.6185	1299.6112	6.1409e+002	1299.6207	-0.0095	-7.3091
28	y13	1399.6889	1398.6816	1.4667e+002	1398.6892	-0.0075	-5.3722

31	y14	1500.7403	1499.7330	2.2031e+002	1499.7368	-0.0038	-2.5658
34	y15	1601.7865	1600.7792	3.3676e+002	1600.7845	-0.0053	-3.2959
17	y21	1151.0598	2300.1051	1.2364e+002	2300.1284	-0.0234	-10.1564
23	y23	1257.0959	2512.1772	3.1675e+003	2512.2081	-0.0309	-12.2987
26	y24	1313.6408	2625.2669	9.9421e+002	2625.2922	-0.0252	-9.6100
27	y25	1378.1655	2754.3164	5.3691e+002	2754.3348	-0.0184	-6.6735
29	y26	1428.6902	2855.3659	1.1222e+003	2855.3824	-0.0165	-5.7887
30	y27	1486.2053	2970.3960	1.2642e+003	2970.4094	-0.0134	-4.5169
33	y28	1559.7272	3117.4398	1.4823e+003	3117.4778	-0.0380	-12.1852
36	y29	1624.2583	3246.5020	9.8356e+002	3246.5204	-0.0184	-5.6651
39	y30	1688.7694	3375.5243	1.2550e+003	3375.5630	-0.0387	-11.4499
45	y31	1753.2904	3504.5662	1.5234e+003	3504.6056	-0.0393	-11.2278
55	y32	1809.8250	3617.6354	9.9857e+002	3617.6896	-0.0542	-14.9808
57	y33	1845.3571	3688.6996	8.6278e+002	3688.7267	-0.0272	-7.3651
59	y34	1910.8620	3819.7095	8.7020e+002	3819.7672	-0.0577	-15.1161
57	y34	1845.3571	3688.6996	8.6278e+002	3819.7672	0.0584	15.2878
62	y35	1960.4187	3918.8228	3.0574e+002	3918.8356	-0.0129	-3.2831
42	y75	1705.8080	8524.0035	4.9277e+002	8655.1863	-0.0568	-6.5575

Protein name: Acid stress chaperone HdeB (Uniprot accession no. P0AET2)

Charge state: +5

Observed monoisotopic mass: 9057.3468 Da

Sequence:
ANESAKDMTCQEFIDLNPKAMTPVAWWMLHEETVYKGGDTVTLNETDLTQIPKVIEYCKKNPQKNLYTFKNQASNDLPN

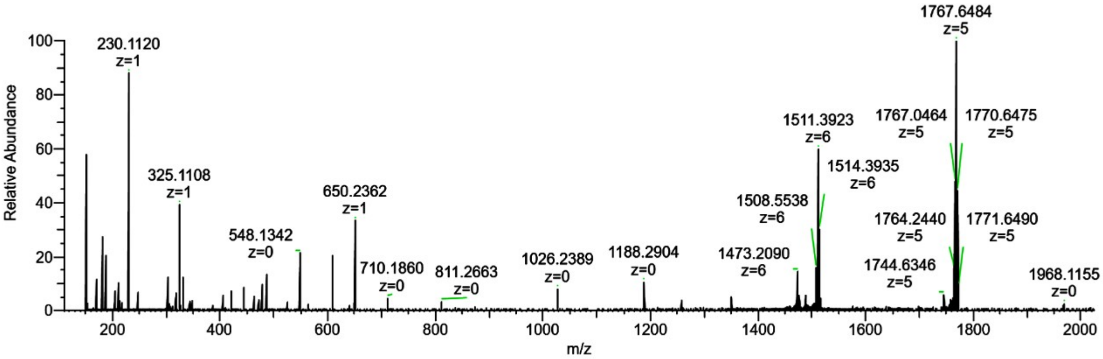

ID	Name	m/z Monoisotopic	Mass Monoisotopic	Intensity	Theoretical Mass	Error (Da)	Error (ppm)
10	b7	716.3205	715.3132	1.2522e+002	715.3137	-0.0005	-0.6599
37	b67	1542.9328	7709.6276	1.8229e+002	7711.7485	0.0060	0.7797
38	b68	1563.1545	7810.7363	1.0007e+002	7812.7962	0.0670	8.5722
41	b74	1698.2065	8485.9963	1.8497e+002	8488.1302	-0.0070	-0.8301
43	b75	1721.0329	8600.1280	3.9333e+002	8602.1731	0.0818	9.5097
48	b76	1744.0288	8715.1075	2.6939e+003	8717.2001	0.0343	3.9324
25	b76	1453.5261	8715.1130	1.0166e+003	8717.2001	0.0398	4.5699
54	b77	1766.6602	8828.2647	8.1789e+003	8830.2841	0.1074	12.1683

Protein name: Phosphocarrier protein HPr (Uniprot accession no. P0AA04)

Charge state: +6

Observed monoisotopic mass: 9113.6642 Da

Sequence:
MFQQEVTITAPNGLHTRPAAQFVKEAKGFTSEITVTSNGKSASAKSLFKLQTLGLTQGTVVTISAEGEDEQ
KAVEHLVKLMAELE

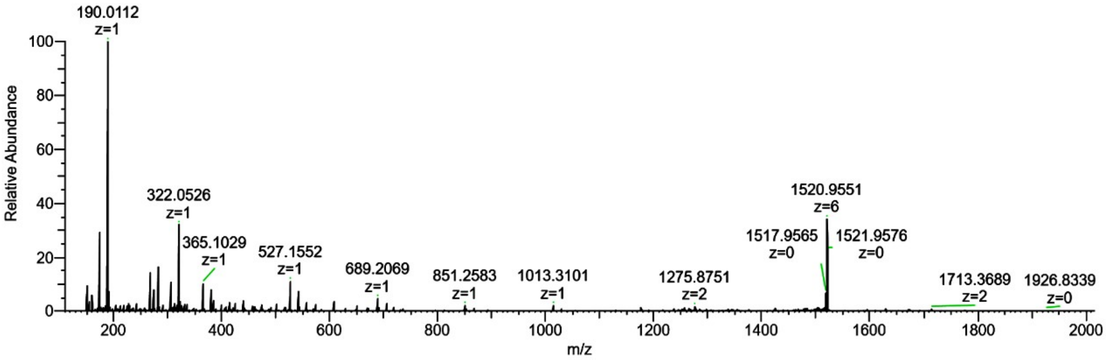

ID	Name	m/z Monoisotopic	Mass Monoisotopic	Intensity	Theoretical Mass	Error (Da)	Error (ppm)
45	b53	1423.4911	5689.9353	7.9261e+002	5689.9930	-0.0577	-10.1417
45	b53	1423.4911	5689.9353	7.9261e+002	5689.9930	0.0153	2.6887
54	b84	1495.4396	8966.5941	5.4121e+002	8966.6840	-0.0899	-10.0289
54	b84	1495.4396	8966.5941	5.4121e+002	8966.6840	-0.0169	-1.8871
75	y32	1712.8609	3423.7073	3.3188e+002	3423.7442	-0.0370	-10.7955
75	y32	1712.8609	3423.7073	3.3188e+002	3423.7442	0.0360	10.5276
73	y77	1628.4404	8137.1658	6.8800e+002	8137.2684	-0.1026	-12.6107
73	y77	1628.4404	8137.1658	6.8800e+002	8137.2684	-0.0296	-3.6391

Protein name: Phosphate starvation-inducible protein PsiF (Uniprot accession no. P0AFM4)

Charge state: +6

Observed monoisotopic mass: 9443.4898 Da

Sequence:
AAERTLTPQQQRMTSCNQQATAQALKGDARKTYMSDCLKNSKSAPGEKSLTPQQQKMRECNNQATQQSLKGDDRNKFMSACLKKAA

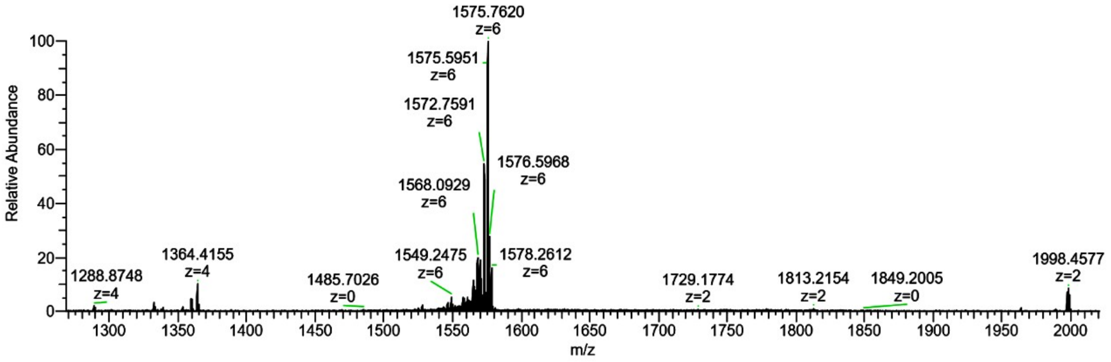

ID	Name	m/z Monoisotopic	Mass Monoisotopic	Intensity	Theoretical Mass	Error (Da)	Error (ppm)
28	b50	1363.6666	5450.6374	9.8024e+002	5450.6551	-0.0177	-3.2455
30	b84	1548.2440	9283.4203	6.0561e+002	9386.5526	0.0153	1.6308
41	y36	1997.4352	3992.8559	6.7765e+002	4095.9823	0.0212	5.1806
26	y36	1331.9713	3992.8920	2.1292e+002	4095.9823	0.0573	13.9993

Protein name: Uncharacterized protein YbgS (Uniprot accession no. P0AAV6)

Charge state: +7

Observed monoisotopic mass: 10464.5632 Da

Sequence:
ADSGAQTNNGQANAAADAGQVAPDARENVAPNNVDNNGVNTGSGGTMLHSDGSSMNNDGMTKD
EEHKNTMCKDGRCPDINKKVQTGDGINNDVDTKTDGTTQ

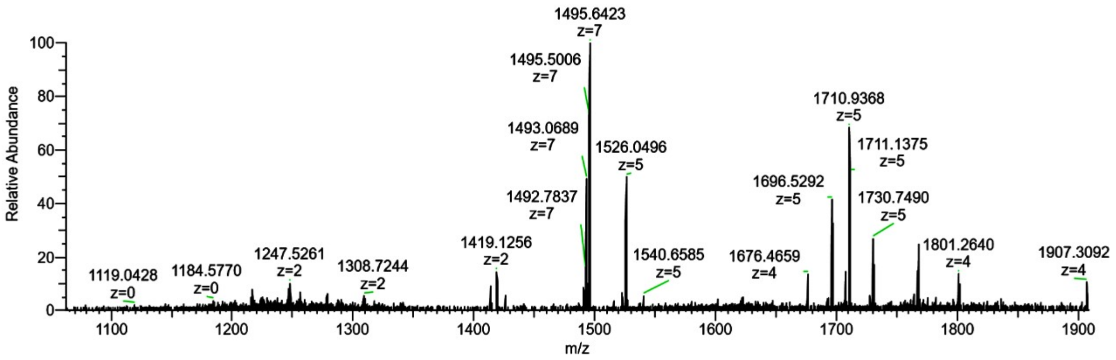

ID	Name	m/z Monoisotopic	Mass Monoisotopic	Intensity	Theoretical Mass	Error (Da)	Error (ppm)
19	y63	1340.7579	6698.7530	2.8682e+002	6699.9170	0.0047	0.6970
25	y68	1440.6092	7198.0095	4.8371e+002	7199.1197	0.0585	8.1286
16	y72	1271.3656	7622.1499	1.1990e+003	7623.3267	-0.0082	-1.0700
29	y72	1525.4392	7622.1596	8.7935e+002	7623.3267	0.0015	0.2028
21	y80	1413.4375	8474.5811	1.0801e+003	8475.7357	0.0141	1.6680
24	y81	1425.2749	8545.6058	1.6254e+003	8546.7728	0.0017	0.1938
26	y82	1441.7882	8644.6857	3.3924e+002	8645.8412	0.0132	1.5240
27	y84	1472.6308	8829.7411	1.9328e+002	8830.9213	-0.0115	-1.3006

Protein name: Protein YgiW (Uniprot accession no. P0ADU5)

Charge state: +7

Observed monoisotopic mass: 11968.8868 Da

Sequence:
AEQGGFSGPSATQSQAGGFQGPNGSVTTVESAKSLRDDTWVTLRGNIVERISDDLYVFKDASGTINVDIDHKRWNGVTVTPKDTVEIQGEVDKDWNSVEIDVKQIRKVNP

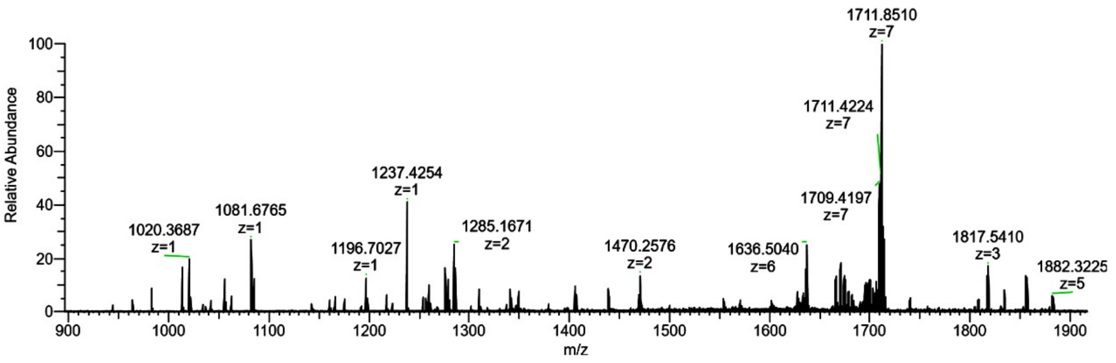

ID	Name	m/z Monoisotopic	Mass Monoisotopic	Intensity	Theoretical Mass	Error (Da)	Error (ppm)
50	b53	1816.5471	5446.6194	1.8482e+003	5488.6519	0.0784	14.2843
52	b54	1854.8721	5561.5945	1.4333e+003	5603.6788	0.0266	4.7479
51	b86	1833.0904	9160.4155	9.9085e+002	9202.5208	0.0056	0.6124
42	b109	1694.4066	11853.7953	1.6044e+003	11895.9344	-0.0282	-2.3713
13	y8	982.6109	981.6036	3.5968e+002	981.6083	-0.0047	-4.8125
16	y9	1081.6793	1080.6721	1.2077e+003	1080.6767	-0.0047	-4.3362
20	y10	1196.6996	1195.6923	5.6489e+002	1195.7037	-0.0114	-9.5007
31	y12	1438.8373	1437.8300	4.3514e+002	1437.8303	-0.0003	-0.2267
12	y16	963.0268	1924.0390	2.3372e+002	1924.0530	-0.0140	-7.2732
17	y18	1084.5784	2167.1422	8.5722e+002	2167.1749	-0.0327	-15.0874
27	y22	1284.6682	2567.3219	1.8753e+003	2567.3343	-0.0124	-4.8284
29	y23	1348.6936	2695.3727	6.1842e+002	2695.3929	-0.0202	-7.4928
30	y24	1405.2291	2808.4436	6.7897e+002	2808.4770	-0.0334	-11.8890
32	y25	1469.7650	2937.5154	9.7927e+002	2937.5195	-0.0042	-1.4260
34	y28	1627.3230	3252.6314	5.0534e+002	3252.6626	-0.0312	-9.5777

18	y30	1160.2642	3477.7707	3.1478e+002	3477.8103	-0.0396	-11.3755
19	y40	1165.1019	4656.3785	5.2075e+002	4656.4412	-0.0627	-13.4581
33	y40	1553.1375	4656.3907	4.5782e+002	4656.4412	-0.0504	-10.8304
36	y87	1635.6608	9807.9212	3.9908e+003	9808.0411	-0.1199	-12.2243

Protein name: Acid stress chaperone HdeA (Uniprot accession no. P0AES9)

Charge state: +5

Observed monoisotopic mass: 11968.8868 Da

Sequence:
ADAQKAADNKKPVNSWTCEDFLAVDESFQPTAVGFAEALNNKDKPEDAVLDVQGIATVTPAIVQACTQDKQANFKDKVKGEWDKIKKDM

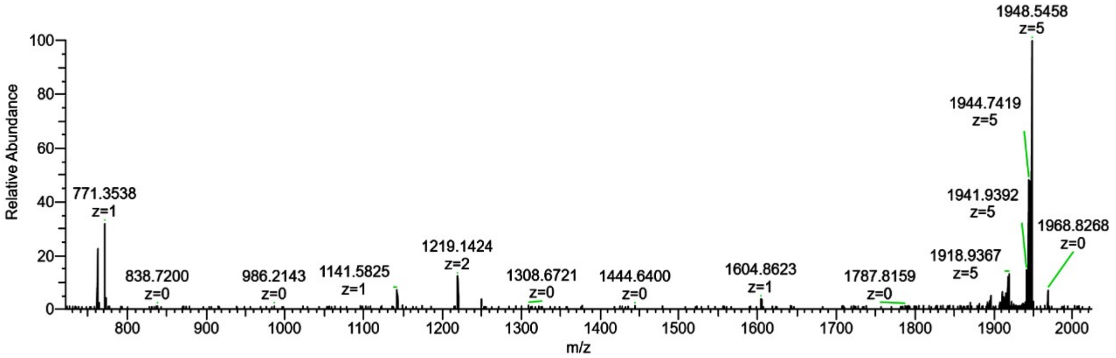

ID	Name	m/z Monoisotopic	Mass Monoisotopic	Intensity	Theoretical Mass	Error (Da)	Error (ppm)
3	b8	771.3584	770.3511	1.6472e+002	770.3559	-0.0048	-6.1984
18	b83	1795.2445	8971.1862	3.5522e+002	8973.3816	-0.0643	-7.1633
17	y81	1793.8864	8964.3956	4.5582e+002	8964.4727	-0.0772	-8.6068
5	y87	1592.1034	9546.5769	1.0901e+003	9548.7645	-0.0565	-5.9144

Protein name: Superoxide dismutase [Mn]

Charge state: +14

Observed monoisotopic mass: 49758.6193 Da (dimer)

Sequence(monomer):

SYTLPSLPYAYDALEPHFDKQTMEIHHTKHHQTYVNNANAALESLPEFANLPVEELITKLDQLPADKKTVL
RNNAGGHANHSLFWKGLKKGTTLQGDLKAAIERDFGSVDNFKAEFEKAAASRFGSGWAWLVLKGDKL
AVVSTANQDSPLMGEAISGASGFPIMGLDVWEHAYYLKFQNRRPDYIKEFWNVVNWDEAAARFAAKK

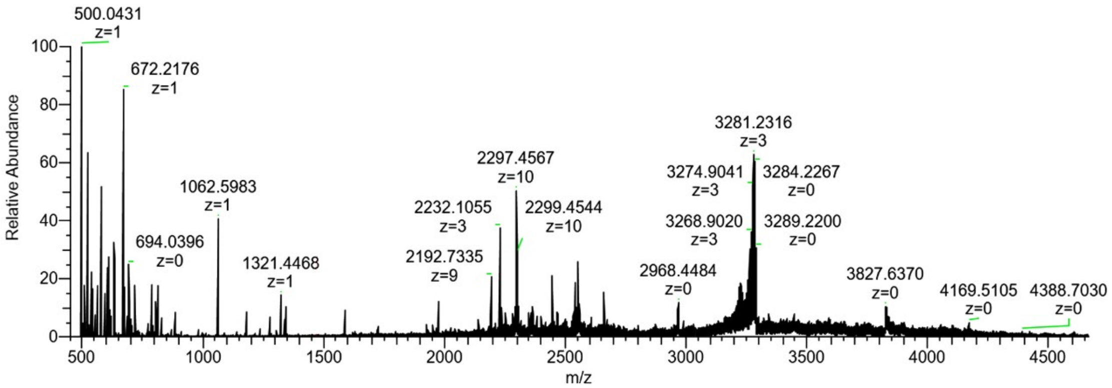

ID	Name	m/z Monoisotopic	Mass Monoisotopic	Intensity	Theoretical Mass	Error (Da)	Error (ppm)
3	y10	1062.6039	1061.5966	8.5366e+001	1061.5981	-0.0015	-1.4280
4	y38	1587.1288	4758.3645	3.8800e+001	4758.4036	-0.0391	-8.2189
6	y58	2230.7496	6689.2271	1.9595e+002	6689.3204	-0.0933	-13.9503
20	y205	2551.1626	22951.3980	2.7072e+002	22951.6094	-0.2114	-9.2094

Antigen 43 a

Charge state: +14

Observed monoisotopic mass: 46038.1400 Da

Sequence:

ADIVVHPGETVNGGTLANHDNQIVFGTTNGMTISTGLEYGPDNEANTGGQWVQDGGTANKTTVTSG
GLQRVNPGGSVSDTVISAGGGQSLQGRAVNTTLNGGEQWMHEGAIATGTVINDKGWQVVKPGTVAT
DTVVNTGAEGGPDAENGDTGQFVRGDAVRTTINKNGRQIVRAEGTANTTVVYAGGDQTVHGHALDT
TLNGGYQYVHNGGTASDTVVNSDGWQIVKNGGVAGNTTVNQKGRLQVDAGGTATNVTLKQGGALV
TSTAATVTGINRLGAFSVVEGKADNVVLENGGRLDVLTGHTATNTRVDDGGTLDVRNGGTATTVSMG
NGGVLLADSGAAVSGTRSDGKAFSIGGGQADALMLEKGSSFTLNAGDTATDTTVNGGLFTARGGTLAG
TTTLNNGAILTLSGKTVNNDTLTIREGDALLQGGSLTGNGSVEKSGSGTLTVSNTTLTQKAVNLNEGTLTL
NDSTVTTDVIAQRGTALKLTGSTVLNGAID

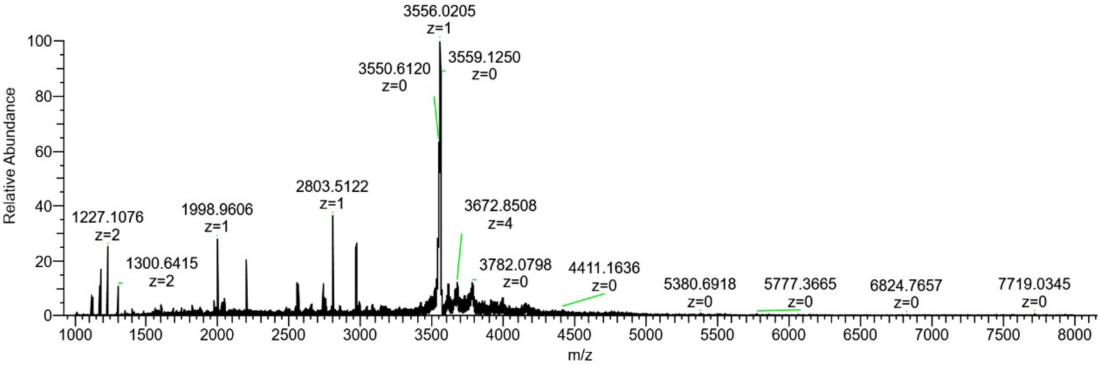

ID	Name	m/z Monoisotopic	Mass Monoisotopic	Intensity	Theoretical Mass	Error (Da)	Error (ppm)
5	b20	1997.9440	1996.9368	7.5395e+001	1996.9602	-0.0234	-11.7413
1	b22	1120.5307	2239.0468	1.9331e+001	2239.0617	-0.0150	-6.6876
2	b23	1177.0733	2352.1321	4.3135e+001	2352.1458	-0.0137	-5.8274
3	b24	1226.6008	2451.1870	7.1793e+001	2451.2142	-0.0272	-11.0921
4	b25	1300.1306	2598.2467	2.7690e+001	2598.2826	-0.0359	-13.8060
14	b176	2967.8226	17800.8917	2.0446e+002	17800.6580	0.2337	13.1314
6	y22	2198.2352	2197.2279	4.9849e+001	2197.2430	-0.0151	-6.8654
13	y28	2802.5255	2801.5182	1.0687e+002	2801.5134	0.0048	1.7051

[1] K.I. Kocurek, J. Havlikova, E. Buchan, A. Tanner, R.C. May, H.J. Cooper, Electroporation and Mass Spectrometry: A New Paradigm for In Situ Analysis of Intact Proteins Direct from Living Yeast Colonies, Analytical Chemistry 92 (2020) 2605-2611.
[2] J. Havlikova, R.C. May, I.B. Styles, H.J.J.J.o.t.A.S.f.M.S. Cooper, Liquid Extraction Surface Analysis Mass Spectrometry of ESKAPE Pathogens, (2021).
